# The threat of gambling to public health in Ghana: time to act

**DOI:** 10.1177/17579139241229886

**Published:** 2024-08-06

**Authors:** E Badu, G Crawford, J Hallett, D Vujcich, ME Bellringer

**Affiliations:** Collaboration for Evidence, Research and Impact in Public Health, School of Population Health, Curtin University, Perth, WA, Australia; Collaboration for Evidence, Research and Impact in Public Health, School of Population Health, Curtin University, Room 453 Building 400, Curtin UniversityKent Street, Bentley Western Australia 6102, Australia; Collaboration for Evidence, Research and Impact in Public Health, School of Population Health, Curtin University, Perth, WA, Australia; Collaboration for Evidence, Research and Impact in Public Health, School of Population Health, Curtin University, Perth, WA, Australia; Gambling and Addictions Research Centre, School of Public Health and Interdisciplinary Studies, Faculty of Health and Environmental Sciences, Auckland University of Technology, Auckland, New Zealand

*In this manuscript, Badu and co-authors provide a contemporary picture of gambling and gambling harm in Ghana and argue for a comprehensive public health response to prevent and minimise these. This article works towards expanding the international literature on gambling in low-income countries, which is a global health priority.*

## Context

Like many other sub-Saharan African countries, Ghana has yet to adopt a public health response to the growing threat of gambling and the gambling industry. Currently, there is no national programme aimed at preventing and minimising gambling harm and effectively regulating the gambling industry. The rhetoric from industry and government is centred on ‘responsible gambling’, which frames gambling and its harms as an issue of personal responsibility and positions the individual as the sole architect of their behaviour.^1^ However, as the recent Lancet editorial on the commercial determinants of health has argued, ‘commercial actors are diverse and many play a vital role in society, but the products and practices of many are having increasingly negative impacts on human and planetary health and equity’.^2^

The expansion of the gambling industry into sub-Saharan Africa has intensified in the last decade driven by rapid technological advancements and the introduction of tighter gambling regulations in high-income countries.^1^ The prevailing weak and outdated gambling regulatory regime across many countries in the region have enabled large transnational gambling brands to adapt their existing platforms to attract new customers and grow their market share, as the industry looks for new sources of profit.^3^ At present, there is less research about gambling industry activities and influence on gambling behaviour and harms in sub-Sahara Africa.^4^ However, the predatory practices of transnational tobacco,^5^ alcohol^6,7^ and food^8,9^ industries are well documented and offer useful insights into how the gambling industry may be employing similar tactics. These include sophisticated marketing, enhanced corporate imaging through corporate social responsibility, exploitation of the acute need for sponsorships, misconceptions about the gambling industry contribution to employment and revenue for government, alliances with politicians and circumventing weak regulations to drive the uptake of gambling and gambling harm.

The primary objective of the gambling industry is to grow and sustain profits for their shareholders, not for the consumers.^10^ To achieve their objective, gambling products are aggressively marketed, often to young people, and positioned as a legitimate and enjoyable means to make quick money.^1^ Targeting young people is a deliberate tactic of the industry to build future loyalists and consumers of gambling products.^11^ Growing profits and market share is therefore at the expense of often the most deprived and vulnerable people in society, and the misconception of revenue generated by the government is built on the impoverishment of people who are predominantly unemployed and living in precarious conditions.^10^

**Figure:**
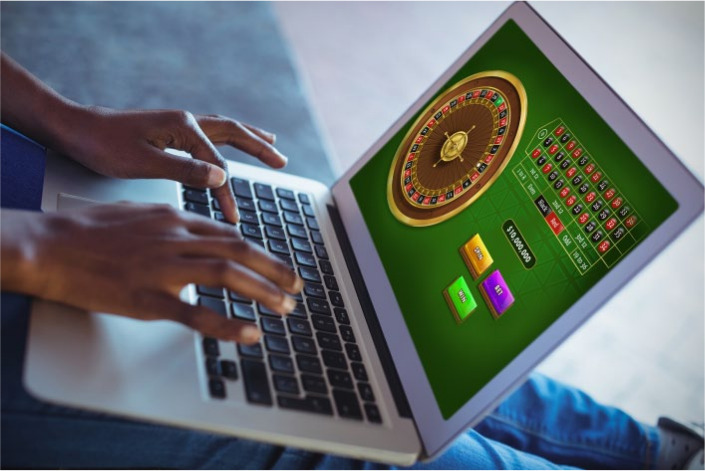


Many gambling activities legally operate in Ghana including lotteries, casinos, horse racing and sports betting.^12^ The available evidence suggests sports betting may be the most popular gambling activity and poses the biggest threat to the health of children, young adults and vulnerable communities.^13,14^ A strong sporting culture likely fuels the availability of sports betting along with a significant support base for sports (especially European football), high mobile phone use and the availability of a mobile payment system infrastructure, enabling stakes to be placed via phones and wins to be paid promptly.^1,15^

This conducive environment, coupled with weak advertising regulations and poor operator monitoring, has created favourable conditions for the gambling industry to rapidly expand its products and profits at the expense of the public’s health and wellbeing, particularly those most vulnerable – children and younger adults.

## Who is at Risk and what are the Impacts?

Studies from Ghana have reported common reasons for participating in gambling, including winning money, leisure, socialisation and unmet psychological and social needs.^16,17^ While the extent of gambling participation and the prevalence of gambling harm in Ghana are unknown, available studies indicate gambling participation is relatively prevalent among vulnerable groups such as children. For instance, a nationwide study of 5,024 school children aged 8–17 years reported a gambling prevalence rate of 3.1%, with higher rates reported among males.^13^ Another study of 1,101 randomly selected in-school adolescents aged 10–19 years living in a rural area reported a problem gambling prevalence of 34.3%, higher among males than females.^14^ We anticipate that the consequences of gambling to children, young adults and other vulnerable groups could be far more significant than currently reported.

As an activity involving monetary losses and high risks, the consequences of gambling including costs to health and social systems far outweigh the benefits to individuals, their families and communities. Specifically, studies in Ghana have found gambling linked to financial stress (losing money, people incurring debts they are unable to repay, stealing to stake bets), poor mental health and wellbeing (depression, mental instability, sleepless nights), poor academic performance and challenges with personal and social life including family breakdown and crime.^16,18^

## Why is Public Health Action Urgently Needed?

A comprehensive public health approach to gambling and its harms is vital and has been framed as twofold.^19^ First, it recognises that the drivers of gambling harms result from the interrelationship of commercial, political, economic, social and behavioural determinants.^19^ Second, it champions evidence-informed policies and strategies, free from industry influence, that prioritise and safeguard the health and wellbeing of individuals and populations from harm caused by the gambling industry and its products.^19^ This framework provides a useful starting point to guide public health action on the prevention of gambling and its harms in Ghana.

We have insufficient data on the profiles of those who gamble or how and what marketing strategies encourage betting in Ghana. While the use of celebrities for alcohol advertising has been banned, this is not the case for gambling. For example, television gambling advertising features prominent Ghanaian athletes, football team shirts display gambling logos, and the gambling industry is currently the headline sponsor of the Ghana Premier League. These tactics have far-reaching impacts on children and young people who see players they idolise normalising these harmful products and perpetuating a new culture of modernity and legal and appealing means of making money.^11^ The trend towards rapid gambling uptake will not change unless gambling marketing is effectively regulated with strict enforcement of regulations on advertising material size and placement.^20^ In addition, there is a need for evidence-informed, well-resourced social marketing-based public education campaigns to counter gambling advertising and the use of influential sports stars. Examining effective public health strategies previously used to limit the influence of other harmful industries and products will be instructive.

People have a choice in how they spend their money. However, governments can and must play an essential stewardship role in protecting people by creating supportive and safe environments free from the influence and impact of harmful products and industries, particularly children and vulnerable populations. In 2006, Ghana enacted the Gaming Act 2006 (Act 721). The act consolidated all the laws relating to gambling activities (other than lotteries) and established the Gaming Commission to regulate, control, monitor and supervise the gambling operation in Ghana. However, the current law and the commission do not adequately address contemporary gambling issues and contexts, such as online gambling. The law primarily focuses on ensuring fair and equitable participation in the gambling market by gambling operators and with minimal set of protections for people who engage in gambling. It does not set out an overarching objective to protect the public’s health nor extends current protections to families, relatives and communities of the people who engage in gambling. Per the Act, the commission is required to report on the number of licences issued and maintain a licence register. However, there is no requirement to produce reports on gambling participation levels, revenues generated or funding disbursed for social responsibility, including research or community development. We argue these should be key mandates of the commission in the current environment. The law and the commission will benefit from a structural review underpinned by public health principles to effectively address the rapidly evolving gambling landscape and its threat to the public’s health.

## Conclusion

We must act on the public health of Ghana now by demonstrating leadership in monitoring industry tactics and exerting pressure on decision-makers to regulate the gambling industry effectively. Emphasis must shift from personal responsibility and individual harms to action on the economic, commercial and political determinants by investigating and addressing how the gambling industry influences young and vulnerable people into participating in and profiting from their harmful products. Practical next steps include raising community and political awareness about the harms of gambling, demanding an urgent review of the Gaming Act 2006 (Act 721), reducing gambling marketing with a focus on children’s exposure and strengthening the commission’s purview to oversee gambling operators effectively. There is a finite window of opportunity to slow and reverse gambling harms – that time is now.

## References

[1] Glozah F Bunn C Sichali J , et al. Young people and gambling in sub-Saharan Africa: towards a critical research agenda. J Br Acad 2023;11:153–72.

[2] The Lancet. Unravelling the commercial determinants of health. Lancet 2023;401:1131. 36966781 10.1016/S0140-6736(23)00590-1

[3] Reith G Wardle H Gilmore I . Gambling harm: a global problem requiring global solutions. Lancet 2019;394:1212–4. 10.1016/S0140-6736(19)31991-9 31443927

[4] Bitanihirwe BKY Adebisi T Bunn C , et al. Gambling in Sub-Saharan Africa: traditional forms and emerging technologies. Curr Addict Rep 2022;9(4):373–84. 10.1007/s40429-022-00449-0 PMC9595076 36312763

[5] Amul GGH Tan GPP van der Eijk Y . A systematic review of tobacco industry tactics in Southeast Asia: lessons for other low- and middleincome regions. Int J Health Policy Manag 2021;10:324–37. 10.34172/ijhpm.2020.97 PMC9056152 32610812

[6] Bertscher A London L Orgill M . Unpacking policy formulation and industry influence: the case of the draft control of marketing of alcoholic beverages bill in South Africa. Health Policy Plan 2018;33:786–800. 29931204 10.1093/heapol/czy049

[7] Milsom P Smith R Modisenyane SM , et al. Does international trade and investment liberalization facilitate corporate power in nutrition and alcohol policymaking? Applying an integrated political economy and power analysis approach to a case study of South Africa. Global Health 2022;18:32. 35279184 10.1186/s12992-022-00814-8 PMC8917365

[8] Boachie MK Goldstein S Kruger P , et al. Beverage industry’s advertising expenditures and airtimes in South Africa from 2013 to 2019 target children and families. J Public Health Res 2023;12(2):22799036231168207. 37122639 10.1177/22799036231168207 PMC10134126

[9] Abdool Karim S Kruger P Hofman K . Industry strategies in the parliamentary process of adopting a sugar-sweetened beverage tax in South Africa: a systematic mapping. Global Health 2020;16:116. 33302993 10.1186/s12992-020-00647-3 PMC7725882

[10] Rae M Fell G . Protecting the Public From Being Harmed or Exploited by Gambling and the Gambling Industry. Available online at: https://www.adph.org.uk/2022/06/protecting-the-public-from-being-harmed-or-exploited-by-gambling-and-the-gambling-industry/

[11] Thomas SL van Schalkwyk MCI Daube M , et al. Protecting children and young people from contemporary marketing for gambling. Health Promot Int 2023;38:daac194. 10.1093/heapro/daac194 PMC10024482 36932993

[12] Gaming Commission of Ghana. Licensed operators, 2023. https://gamingcommission.gov.gh/license_operators.php

[13] Kyei-Gyamfi S Coffie D Abiaw MO , et al. Prevalence, predictors and consequences of gambling on Children in Ghana. BMC Public Health 2022;22:2248. 36460997 10.1186/s12889-022-14750-0 PMC9719180

[14] Odame SK Quarshie EN Oti-Boadi M , et al. Adolescent problem gambling in rural Ghana: prevalence and gender differentiation. J Gambl Stud 2021;37(1):83–105. 33179195 10.1007/s10899-020-09987-6 PMC7882566

[15] Akanle O Fageyinbo KT . European football clubs and football betting among the youths in Nigeria. Soccer & Society 2019;20:1–20.

[16] Amoah-Nuamah J Agyemang-Duah W Mensah B , et al. University students’ reasons and attitudes towards online gambling and its implication on their lives. J Gambl Stud 2023;39:203–24. 10.1007/s10899-022-10143-5 35804279

[17] Tagoe VNK Yendork JS Asante KO . Gambling among youth in contemporary Ghana: understanding, initiation, and perceived benefits. Africa Today 2018;64:52–68.

[18] Acheampong EY Sarpong EO Mahamah M . Understanding sports betting among young male student-teachers in Ghana. J Gambl Issues 2022;49:174–200.

[19] Thomas SL Crawford G Daube M , et al. Time for policies on gambling to benefit health – not the gambling industry. Health Promot J Austr 2023;34:267–71. 10.1002/hpja.721 37038275

[20] Bouguettaya A Lynott D Carter A , et al. The relationship between gambling advertising and gambling attitudes, intentions and behaviours: a critical and meta-analytic review. Curr Opin Behav Sci 2020;31:89–101.

